# Therapeutic efficacy and immunoregulatory effect of Qiangji Jianli Capsule for patients with myasthenia gravis: Study protocol for a series of randomized, controlled N-of-1 trials

**DOI:** 10.1097/MD.0000000000023679

**Published:** 2020-12-18

**Authors:** Senhui Weng, Zhixin Fan, Guoyu Qiu, Fengbin Liu, Linwen Huang, Jinghao Li, Xiaotao Jiang, Zhixuan Song, Yuxia Gao, Zhuotai Zhong, Long He, Liping Kang, Yunlong Wu, Benshu Chen, Qilong Jiang

**Affiliations:** a Department of Spleen-Stomach, First Affiliated Hospital of Guangzhou University of Chinese Medicine; b Guangzhou University of Chinese Medicine, Guangzhou, China.

**Keywords:** myasthenia gravis, N-of-1 trials, Qiangji Jianli Capsule, TCM

## Abstract

**Introduction:**

Myasthenia gravis (MG) is an autoimmune disease in which antibodies directly target components of the neuromuscular junction, causing neuromuscular conduction damage that leads to muscle weakness. The current pharmaceutical treatment for MG is still not ideal to address the problems of disease progression, high recurrence rate, and drug side effects. Clinical observations suggest that traditional Chinese medicine (TCM) can strengthen immunity and improve symptoms of MG patients, delay the progression of the disease, reduce or even prevent the need for immunosuppressive therapy when used in combination with acetylcholinesterase inhibitors or low-dose prednisone, as well as improve the quality of life of patients. The Qiangji Jianli Capsule (QJC) is a combination of medicinal herbs which is used in traditional Chinese medicine. Since MG is a rare disorder, randomized controlled trials comparing large cohorts are difficult to conduct. Therefore, we proposed to aggregate data from a small series of N-of-1 trials to assess the effect of the Chinese medical prescription QJC, which strengthens the spleen and nourishes Qi, as an add-on treatment for MG with spleen and stomach Qi deficiency syndrome.

**Methods and analysis:**

Single-center, randomized, double-blind, multiple crossover N-of-1 studies will compare QJC versus placebo in 5 adult MG patients with spleen and stomach Qi deficiency syndrome. Patients will undergo 3 cycles of two 4-week intervention periods. According to the treatment schedule, patients will continue to be treated with pyridine bromide tablets, prednisone acetate, tablets and/or tacrolimus capsules throughout the entire trial. Each period consisting of 4-week oral add-on treatment with QJC will be compared with 4-week add-on treatment with a placebo. The primary endpoints are quantitative myasthenia gravis (QMG) test; measurement of the amount of T_reg_ cells and cytokines such as interferon-γ (IFN-γ), interleukin-4 (IL-4), interleukin-17A (IL-17A), and transforming growth factor-β (TGF-β); and corticosteroid or immunosuppressive agent dosage. Secondary outcome measures: Clinical: Evaluation of the effect of TCM syndromes; MG-activities of daily living (MG-ADL) scales; adverse events.

**Ethics and dissemination:**

This study was approved by The First Affiliated Hospital of Guangzhou University of Chinese Medicine (GZUCM), No. ZYYECK[2019]038. The results will be published in a peer-reviewed publication. Regulatory stakeholders will comment on the suitability of the trial for market authorization and reimbursement purposes. Trial registration: Chinese Clinical Trial Register, ID: ChiCTR2000033516. Registered on 3 June 2020, http://www.chictr.org.cn/showprojen.aspx?proj=54618.

## 1 Introduction

Myasthenia gravis (MG), with an approximate annual incidence of 8 to 10 cases per million persons per year,^[1]^ is a rare and intractable autoimmune disease in which antibodies directly target components of the neuromuscular junction, causing neuromuscular conduction damage that leads to muscle weakness.

The pathogenesis of MG still remains completely unclear, but imbalance in immune regulation is considered to be a major contributor.^[2]^ Recent studies have shown that immune cells, especially regular T cells (T_reg_), play an important role in the pathogenesis and progression of MG, and CD4+CD25+T_reg_ phenotypes and functional defects may also be related to its pathogenesis.^[3,4]^ The deletion of self-reactive T cells is responsible for the development of central tolerance in the thymus, where T cells mature. Defective T_reg_ cells are thought to be involved in MG initiation or progression because self-reactive T cells that escape central tolerance are normally controlled by T_reg_ cells and through peripheral tolerance.^[5]^

Currently, western drug treatment of MG involves acetylcholinesterase inhibitors (AChEIs)^[6,7]^ and immunosuppression with high doses of corticosteroids (prednisone), azathioprine, or other immunosuppressant agents; however, these drugs do not reduce the probability of relapse.^[8]^ These can lead to moderate or serious side effects such as osteoporosis, cardiac arrhythmia, and hypotension. A significant advance would be to determine more effective treatments with fewer side effects that reduce the recurrence rate of MG. Fortunately, in China, traditional Chinese medicine (TCM) as a means of treating MG has achieved curative effects. According to the TCM theory, muscles are governed by the spleen, and the Qi of the spleen and stomach can transport water and grain essences absorbed in the gastrointestinal tract to the muscles, making them stronger. As in all syndromes of muscle weakness, MG is generally diagnosed as “flaccidity syndrome” and “Qi-deficiency pattern”, which means that strategies to strengthen the spleen and nourish Qi are vital in the management of MG. Professor Deng Tie-tao, who is the late master of TCM, suggested that ineffective function of the spleen and stomach is the main cause of MG.^[9]^ Professor Deng has been using drastic spleen-stomach supplementation to treat MG since the 1960s. Strengthening spleen and nourishing Qi is highly effective in the treatment of MG, and for this, he created Qiangji Jianli Fang (QJF), a TCM prescription, to treat MG. This prescription is modified from Buzhong Yiqi decoction including *Radix astragali* (*Huangqi*), *Radix codonopsis pilosulae* (*Danshen*), *Atractylodes macrocephala* (*Baizhu*), *Cimicifugae rhizome* (*Shengma*), *Radix bupleuri* (*Chaihu*), *Radix glycyrrhizae* (*Gancao*), and other medicinal herbs.^[10]^

An in-depth study of the chemical constituents of QJF by domestic and foreign scholars has allowed further understanding of its pharmacological effects and clinical applications. Research has shown that QJF might relieve respiratory muscle weakness by reducing the titer of anti-acetylcholine receptor antibodies (AchRAb) and increasing the amplitude of acetylcholine potential and miniature end plate potential to enhance the motor function of diaphragm.^[11]^

Furthermore, the imbalance between Th17 and T_reg_ cells is an important factor in the pathogenesis of myasthenia gravis.^[12]^ QJF has been reported to have a therapeutic effect in a MG rat model, as it modulates the balance of Th17/T_reg_ lymphocytes by affecting the protein expression of their transcription factors.^[13]^

Our hospital, The First Affiliated Hospital of Guangzhou University of Chinese Medicine (GZUCM), is where the late Professor Deng practiced throughout his career. Under his teams efforts, QJF has been processed into a capsule form named Qiangji Jianli Capsule (QJC) which is more convenient for prolonged use. This capsule has also proven to be effective in clinical applications.^[14,15]^

### 1.1 N-of-1 trials

Due to the risk of bias reported in previous clinical studies, further clinical evidence, and rigorous randomized controlled trials (RCTs) are needed to confirm the effect of QJC treatment. N-of-1 trials, which are more suitable for a disease with low incidence like MG, are multi-cycle, double-blind, placebo-controlled cross-over trials using standardized measures of effect, with randomization order independently generated for each patient. They provide the most robust evidence possible about treatment efficacy in an individual patient.^[16]^

Furthermore, the N-of-1 trial is a clinically efficacious evaluation method that meets the self-regulation individualized treatment philosophy and characteristics of the individual medical treatment in TCM. It eliminates the inference of subjective selection of doctors and individual differences of patients, especially for chronic diseases like MG which require long-term treatment.

Therefore, in this study, N-of-1 trials will be used to verify the effectiveness of QJC in the treatment of MG and explore how QJC affects the levels of T_reg_ cells and cytokines in the blood of MG patients. This will allow us to prove that strengthening the spleen and nourishing Qi is an effective method to treat MG with TCM. Scientifically, it provides reliable evidence-based medical data to help develop TCM treatment of MG.

## 2 Objectives

The main objective of this study is to detect the clinical efficacy and immune regulation effectiveness of add-on treatment with QJC for MG using N-of-1 trials. One of the primary endpoints is therapeutic efficacy of QJC versus placebo on muscle strength and endurance which is measured by QMG score. The effect of QJC versus placebo will be determined by analyzing the levels of T_reg_ cells and the cytokines IL-4, IL-17A, INF-γ, and TNF-β in the blood of MG patients, using flow cytometry. The comparison of T_reg_ cells and cytokines before and after the experiment reflects the relationship between the level of inflammation and the level of disease progression. Furthermore, we wish to determine whether the dosage of Western medicines such as prednisone or immunosuppressive agents can be reduced when used together with QJC.

In addition, we aim to describe the effects of add-on treatment with QJC on secondary outcome measures. The secondary outcome measures include MG-activities of daily living (MG-ADL), which is used to measure perceived psychological status, health, and quality of life and evaluation of the effect of TCM syndromes. We will also record any adverse effects of treatment with QJC.

## 3 Methods

### 3.1 Study design and duration

**Figure 1:**
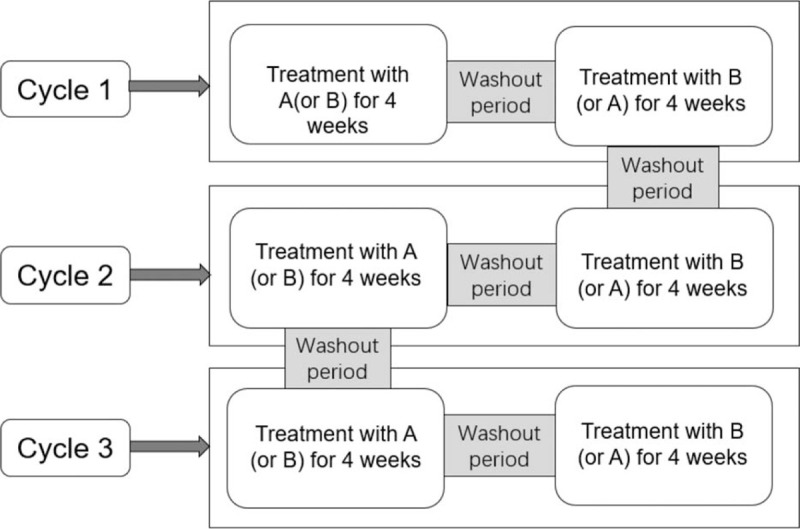
Design of the study. Treatment periods (A and B) will be randomized.

The study will consist of a small series of N-of-1 trials in which each subject can be considered as having his/her own RCT. The design of the trial is presented in Figure 1. The N-of-1 trials will consist of 3 cycles of 8-week duration, each consisting of 4-week for QJC group (A), and 4-week for placebo group(B). Only AB and BA are allowed in each cycle and whether the sequence is AB or BA depends on the SAS random program. Between the 2 treatment periods there will be a 1-week washout period in order to minimize carryover effects between each treatment period and allow us to score and record the outcome. Thus, each N-of-1 trial will take 29 weeks to complete. The protocol conforms to the Standard Protocol Items: CONSORT extension for reporting N-of-1 trials (CENT) 2015.

After multiple cross-over trials have been completed, the researchers will analyze the results of each N-of-1 trial and draft a preliminary report for each patient during week 30 to 31. After blindness is finally unmasked, the treating physician will provide this report to the patient and discuss it with the patient during an outpatient visit in week 30 to 31 and then record the resulting treatment decision.

Appropriately trained medical personnel will carry out the screening for inclusion and exclusion criteria, including enrollment of participants into the N-of-1 trials.

### 3.2 Study population

All MG patients will be enrolled from the First Affiliated Hospital of Guangzhou University of Chinese Medicine (GZUCM). Ethical approval was obtained from the Ethics Committee of The First Affiliated Hospital of GZUCM.

Patients must meet the following inclusion criteria:

1. the patient is diagnosed with MG based on international consensus guidance for management of myasthenia gravis;
2. the patient is in the stable stage of MG requiring hormone treatment, at a dosage of less than 15 mg/day;
3. the patient has a clinical diagnosis of spleen and stomach Qi deficiency syndrome, which is a TCM syndrome diagnosis currently used in clinical practice. Two senior TCM clinicians who assess the patient independently should provide this diagnosis based on the “Criteria of Diagnosis and Therapeutic Effect of TCM Diseases” issued by the State Administration of Traditional Chinese Medicine;^[17]^
4. the patient is age 18 to 65, male or female;
5. the patient can carry out normal speech communication;
6. the patient has signed informed consent for participation.

Patients with the following criteria will be excluded:

1. the patient has muscle weakness that only affects the eye or periocular muscles (MGFA Class I) or who has MG crisis (MGFA Class V).
2. the patient is diagnosed with a major neuropsychiatric disorder (schizophrenia, epilepsy, alcohol abuse, anorexia, and so forth);
3. the patient is diagnosed with cerebrovascular disease, severe liver and kidney dysfunction, or endocrine, urinary, blood system, or other serious primary diseases;
4. the patient is pregnant or planning to have a baby;
5. the patient has allergies to the studied drug and its known ingredients;
6. the patient is participating in other pharmacological clinical trials, or has participated within the past 3 months;
7. the investigator determines the patient is not suitable to take part in the study.

### 3.3 Removal from the trial

Participants will be removed from the study if they meet the following criteria:

- The occurrence of a serious adverse effect (SAE) or suspected unexpected serious adverse reaction (SUSAR);
- A myasthenic crisis or worsening of symptoms requiring any treatment other than the trial medication;
- Patients who voluntarily decide to withdraw from the trial or lose follow-up;
- New exclusion criteria appearing during the study period.

We will still follow up participants who are withdrawn from the study until their planned end of the N-of-1 trial in order to appraise any adverse effects of the trial medication. The cycles which have been finished before a participants withdrawal from the study will be analyzed as part of the trial.

### 3.4 Study setting and recruitment

The trial will be conducted at The First Affiliated Hospital of GZUCM in China using patients recruited from inpatient and outpatient visits.

All clinical measures and tests (e.g. QMG, electrocardiogram (ECG), blood tests) will be performed before the first treatment period and after each 4-week treatment period during the washout period. All other study medications as well as self-reporting questionnaire measures will be administered in the home setting or during an inpatient visit.

### 3.5 Sample size

The calculation of sample size is based on the significance test of the treatment effect in the model. Since the actual number of N-of-1 trials can seldom attain the traditional statistical power consumption level, we used 80% to 90% Monte Carlo simulation to calculate the power consumption of 3, 4, and 5 patients in 3 cycles, which were 0.648, 0.772, and 0.862 respectively.^[18]^ Considering the availability of eligible patients, time, and resources, we selected a sample size of 5 patients and obtained approximately 86% of the power. All treatment outcomes in the study population were measured at the 0.05 level.

### 3.6 Randomization, treatment allocation, and blinding

Patients, treating physicians, and outcome analysts will be blinded to the treatment sequence in the time of the multiple crossover phase of the N-of-1 trials. The orders in which patients receive drugs will be randomized by computer for each individual N-of-1 trial to ensure patient and physician blinding. QJC and placebo treatments will be randomized in a 1:1 ratio per cycle over the 3 cycles of the N-of-1 trial (e.g., AB-BA-BA). Then the 2 drugs together with the randomized medication order will be delivered to a pharmacist. We will use SAS 9.2 statistical software to generate a random number grouping table according to the number of cases and the random proportion. Stratified block group randomization method was used to generate random codes in a 1:1 ratio for the QJC group and the placebo control group. The QJC and placebo have no differences in dosage form, appearance, color, specification, label, and so forth.

The selected block length and random initial seed parameters will be sealed as confidential data. The prepared research drugs will be delivered, stored, and distributed in the First Affiliated Hospital of GZUCM. Patients and investigators will be blinded to all randomization and packaging procedures until completion of the trial.

Unblinding will be allowable when a participant has completed all trials or in the case of SAE, which cannot be treated without knowing what the patient was receiving.

### 3.7 Interventions

During the entire trial, patients treatments with pyridostigmine bromide, low-dose prednisone acetate, and tacrolimus, or other immunosuppressive agents will be continued according to their prestudy treatment schedule. Considering the special flavor and color of Chinese herbs, we chose capsule format called QJC as that is easiest to make similar to placebo. For the N-of-1 trial, oral add-on treatment with QJC (the First Affiliated Hospital of GZUCM, Guangdong medicine system number: Z20070849) will be 5 tablets (1 tablet with 0.5 g medicine) 3 times a day, which will be compared with the add-on treatment with a placebo taken 3 times daily.

For this purpose, we will pour the QJC powders into a non-transparent capsule. The placebo, made by Guangdong Yifang Pharmaceutical Co. Ltd., will be packaged in a non-transparent capsule as well. Both capsules have identical shape, color, and weight.

The doctor will increase or reduce the dose of pyridostigmine bromide, low-dose prednisone acetate, tacrolimus, or other immunosuppressive agents according to the patient's condition. If the disease is not well controlled, prednisolone acetate at 0.5 to 1.0 mg/kg/day or other immunosuppressive drugs may be added to improve the condition. Accountability will be assessed by the dispensing pharmacy, who will maintain records of drugs dispensed. Participant compliance will be assessed by the physician who will ask participants to return all unused study medication and will record pill count. Regular medicine will be documented as concomitant medication.

### 3.8 Care assessment

The following indicators will be measured after each 4-week treatment:

1. Laboratory tests will be performed, including routine blood tests (total number of red cells, hemoglobin volume, erythrocyte specific volume, mean erythrocyte volume, erythrocyte distribution width, mean erythrocyte hemoglobin content, mean erythrocyte hemoglobin concentration, white blood cell count, total number of neutrophils, total number of lymphocytes, total number of monocytes, total number of eosinophils, total basophils, percentage of neutrophils, lymphocyte percentage, percentage of monocytes, eosinophil percentage, basophil percentage, total platelet count, mean platelet volume, platelet distribution width, and specific platelet volume), liver function tests (alanine transaminase, glutamyl transpeptidase), kidney function tests (serum creatinine)
2. Vital signs including blood pressure, respiration rate, heart rate, body temperature, 12-lead electrocardiogram, and physical examination
3. All adverse events occurring during the study, including toxicities and side effects will be reported and recorded in the case report form in detail.

### 3.9 Rescue medication

The protocol does not specify any lifesaving drugs to treat side effects of QJC treatment because, in our experience, these side effects are commonly mild at the dose used in this study (i.e., ulcers, sore throat and any other symptoms of *Shanghuo* (heatiness), which is the unique concept in TCM). If SAEs occur, the patient will be withdrawn from the trial and appropriate treatment will be started.

The fundamental benefit of TCM is syndrome differentiation diagnosis and treatment. In our study, all the patients will be included based on syndrome differentiation and who have a clinical diagnosis of spleen and stomach Qi deficiency syndrome, and side effects of Chinese medicine will rarely ever occur as long as the diagnosis is accurate.

### 3.10 Analysis

We will use SPSS 22.0 software to perform descriptive statistical analysis and difference detection. Measurement data that conforms to normal distribution mean and standard deviation will be evaluated, and those data that do not conform to normal distribution will be expressed as median or mode. Chi-Squared test will be used for comparison between data sets. Covariance analysis/repeated measures analysis of variance (ANOVA) will be used when comparing normal distribution and homogeneity of variance among measurement data sets. Paired rank sum test will be used for non-normal distribution or uneven variance. To ensure the accuracy and integrity of the data, double data entry and proofreading will be performed by 2 independent researchers.

### 3.11 Ethics approval

Our study was accepted by the Ethics Committee of The First Affiliated Hospital of GZUCM, No. ZYYECK [2019]038 and it is registered under Chinese Clinical Trial Register number ChiCTR2000033516, dated June 3, 2020.

### 3.12 Incentives

The QJC and placebo capsules taken during the N-of-1 trial will be provided by the sponsor and the participants have no need to pay for the capsules. Travel costs made by participants for hospital visits and test of relevant indicators and physical check will be reimbursed.

## 4 Discussion

N-of-1 clinical trials incorporate study design and statistical techniques consorted with standard clinical trials, including randomization, washout and crossover periods, and placebo controls. A series of N-of-1 trials can be used for confirming treatment effectiveness in individual patients as well as population treatment effectiveness. By undertaking N-of-1 trials, we can seek out population treatment effects in patients with rare diseases, where parallel-group RCTs may not be feasible owing to small patient numbers. The N-of-1 trial is a clinically efficacious evaluation program that matches up to the self-regulation individualized treatment philosophy and features of individual medical treatment in TCM. It can also make the clinical study of TCM more scientific and can prevent the inference of subjective selection, implementation and measurement prejudice, and individual patient differences, especially for chronic diseases requiring long-term treatment. Because of the crossover nature of the N-of-1 trial, a limiting factor to its widespread use is the requirement for a stable disease course and for the symptomatic, short-acting nature of the investigational treatment. Generalizability of the treatment effect to the entire population may also be limited if there is substantial heterogeneity, especially if the sample size is small.

The outcomes we expect will be that QJC with oral add-on treatment is better than placebo with oral add-on treatment in improving symptoms, reducing inflammation in the body, and improving patients quality of life with high security. Hence, we can popularize a new therapy method combining western medicine with TCM to treat MG. Although only MG patients with spleen and stomach Qi deficiency syndrome can be used to show that QJC is effective for them in this study, we can still conclude that QJC applies to most MG patients, as most are consistent with spleen and stomach Qi deficiency syndrome according to our clinical observations. Even so, we still need to treat based on syndrome differentiation of TCM before we use QJC. It is very important for patients to have accurate diagnosis based on the TCM theory of syndrome differentiation before enrollment, which will make the effect conclusive. So, this study is innovative as a clinical study of TCM and worthy of promotion and exploration in combination with syndrome differentiation, and it will provide additional strong evidence for the value of MG treatments that combine TCM and western medicine.

## Acknowledgments

We thank Scientific Research of Traditional Chinese Medicine Bureau of Guangdong Province for their funding support.

## Author contributions

**Conceptualization:** Senhui Weng, Zhixin Fan, Guoyu Qiu, Qilong Jiang, Fengbin Liu.

**Data curation:** Senhui Weng, Zhixin Fan, Zhuotai Zhong, Liping Kang, Benshu Chen.

**Formal analysis:** Senhui Weng, Zhixin Fan, Guoyu Qiu, Qilong Jiang.

**Investigation:** Senhui Weng, Xiaotao Jiang, Yunlong Wu.

**Methodology:** Senhui Weng, Zhixin Fan, Guoyu Qiu, Linwen Huang, Jinghao Li, Qilong Jiang, Fengbin Liu.

**Project administration:** Senhui Weng, Zhixin Fan, Guoyu Qiu, Qilong Jiang.

**Resources:** Senhui Weng, Zhixin Fan.

**Software:** Senhui Weng, Linwen Huang, Jinghao Li.

**Supervision:** Senhui Weng, Zhuotai Zhong, Long He, Qilong Jiang.

**Validation:** Senhui Weng, Zhuotai Zhong, Long He.

**Visualization:** Guoyu Qiu, Xiaotao Jiang, Zhixuan Song, Yuxia Gao.

**Writing – original draft:** Senhui Weng, Zhixin Fan, Zhixuan Song, Yuxia Gao.

**Writing – review & editing:** Senhui Weng, Zhixin Fan, Guoyu Qiu, Xiaotao Jiang, Zhixuan Song, Yuxia Gao, Zhuotai Zhong, Long He, Liping Kang, Yunlong Wu, Benshu Chen, Qilong Jiang, Fengbin Liu.
